# Implementation Lessons of a Water Insecurity Intervention During a Drought in Mexico

**DOI:** 10.5334/aogh.4758

**Published:** 2025-08-19

**Authors:** Pablo Gaitán-Rossi, Lucía Félix-Beltrán, Ximena García-Ruiz, Sera L. Young

**Affiliations:** 1 Research Center for Equitable Development EQUIDE, Universidad Iberoamericana Mexico City, Mexico City, Mexico; 2 Department of Anthropology, Institute for Policy Research, Northwestern University, Evanston, Illinois, USA

**Keywords:** climate change, water insecurity, drought, implementation science, SDG-6

## Abstract

*Background:* We are early in our understanding of how to effectively implement interventions to mitigate the harm of extreme climate events on human health. We study the actions of the state government in Nuevo Leon, Mexico, which, in 2022, declared a state of emergency due to water shortages resulting from climatic and infrastructural issues.

*Objective:* To document the facilitators and challenges to the rollout of the government’s strategy to mitigate water insecurity, using the EquIR Implementation Science framework. Our analysis focused on the activities of the Ministry of Social Policy, which coordinated emergency activities. The government’s response included water delivery by tanker trucks, installation of household and community cisterns, and distribution of packaged drinking water.

*Methods:* We used three sources of information: gray and academic literature review, government documents, and 10 key-informant interviews.

*Findings:* Facilitators of government actions were the declaration of an emergency as a policy instrument; multi- and inter-sectoral collaboration; the use of pre-existing social and data infrastructure; technical capacity to identify low-income households without water; and flexibility to convert regular activities to emergency response tasks. Salient challenges included citizen discontent about the lack of water; the absence of a preparedness plan; the scarcity of household equipment to store water; initial inefficiencies with water distribution using tanker trucks; difficulties in installing community cisterns in steep terrain; and staff burnout. A positive externality of the response was the improvement of water distribution in informal settlements.

*Conclusions:* As the need to buffer human health from extreme climate events increases, lessons from Mexico about linking climatic events, social policy, and health outcomes can guide strategies in other locations with increasing drought. This case shows how climatic stressors, infrastructure deficiencies, and the population’s coping capacity interact with the government’s actions to shape the impacts of a crisis and its mitigation efforts. Successful mitigation strategies may result from strengthening inter-sectoral collaboration and an evidence-based culture of prevention.

## Background

On February 2, 2022, a few months into the new state administration’s term, the government of Nuevo Leon declared a state of emergency due to widespread ongoing droughts [1], officially acknowledging a period of an unprecedented water crisis affecting agriculture, livestock, industry, and households by reducing water for human consumption and hygiene [2]. In Mexico, a declaration of emergency grants access to federal support if an event threatens the population’s livelihood. It wasn’t until July 2022 that the federal government published a plan to support Nuevo Leon [3]. In the meantime, the state government had to navigate the emergency, extended until September 2022, when coordination mechanisms held their last meeting. The aim of this research is to document, as a case study, the facilitators and challenges to the rollout of the Nuevo Leon government’s strategy to mitigate household water insecurity. This local experience has important global lessons for other cities facing new health and social threats associated with climate change.

### The context of a water scarcity climate emergency

**Figure 1:**
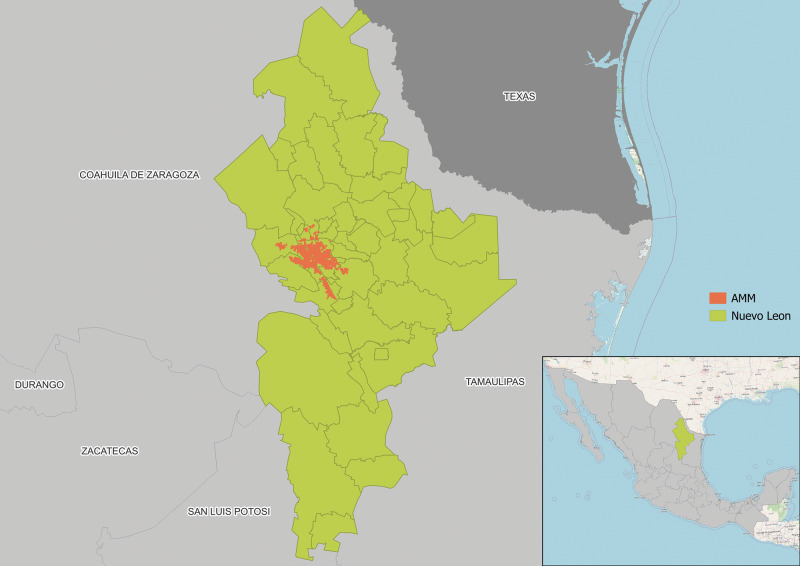
Location of Nuevo Leon in Mexico and its political boundaries.

**Figure 2:**
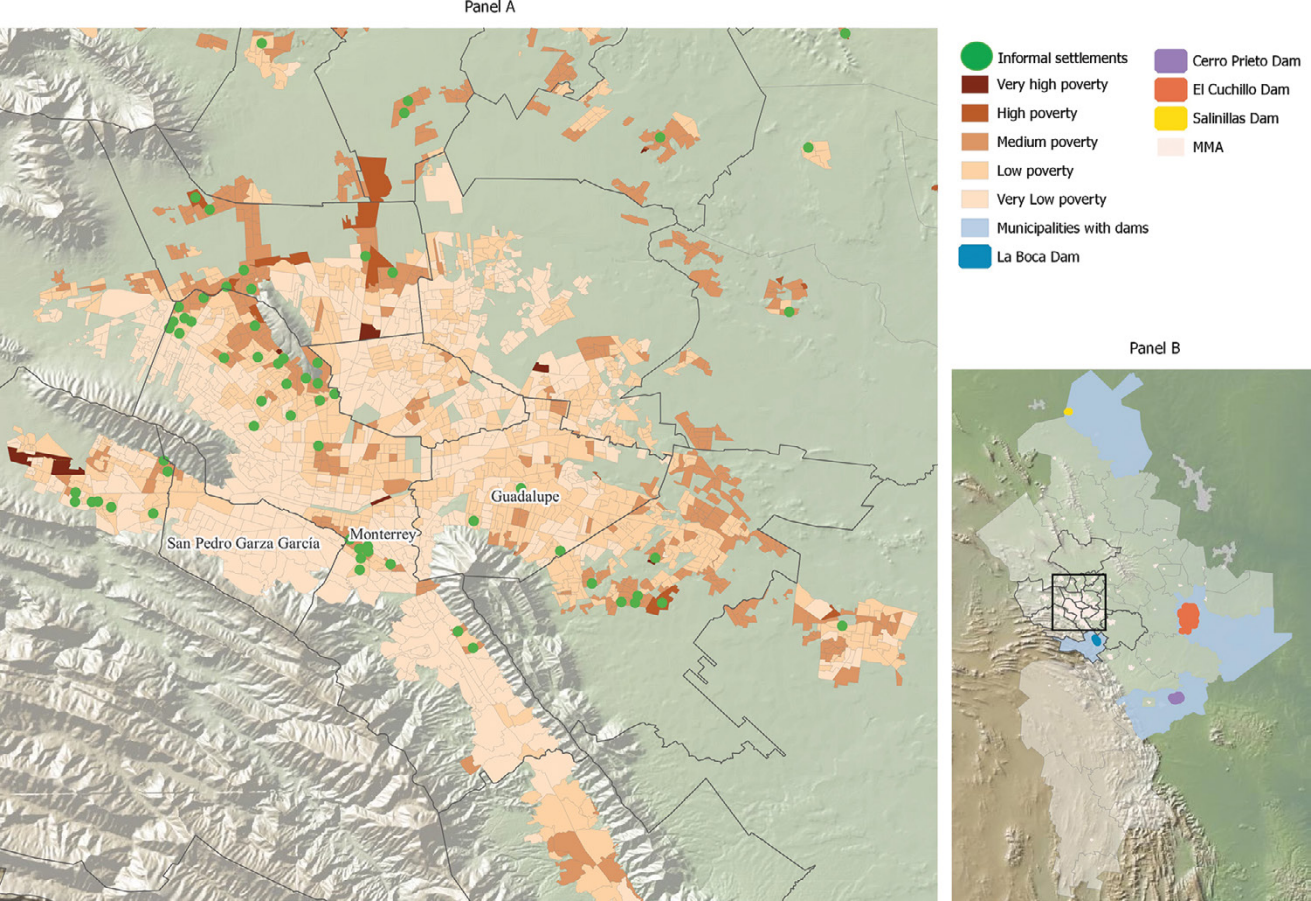
Socioeconomic conditions of municipalities and informal settlements in the Monterrey Metropolitan Area (MMA) in 2022.

Nuevo Leon is an industrial and high-income state in Mexico that borders Texas, USA (See Figure 1). The state has a population of 6 million [4] and the third highest Gross Domestic Product (GDP) per capita in the country [5]. The capital, Monterrey, is the second largest city in Mexico. The Monterrey Metropolitan Area (MMA or AMM in Spanish) concentrates close to 92% of the state’s population [5], who are distributed across eighteen municipalities [6] (see Panel B in Figure 2).

Globally, climate hazards are determined by geographic location and other topographic singularities; however, the burden of climate-related health impacts is higher among the underserved and under-resourced [7]. In Nuevo Leon, according to 2022 official estimates, 16% of the population lived in poverty [8], 22.8% did not have access to health services, and 11.7% experienced food insecurity [8]. In Panel A in Figure 2, brown areas designate municipalities with poverty, which are concentrated on the outskirts. Prior to the declaration of emergency, many households in Nuevo Leon were not equipped to mitigate the effects of water scarcity, as 14.3% did not have domestic water tanks to store water [9]. Overlapping with high poverty areas, in 2022, Nuevo Leon had 154 informal settlements, where people lack access to basic services, including water provision (green dots in Panel A in Figure 2).

Water supply in Nuevo Leon relies on surface and groundwater sources. About 60% of its domestic water comes from three main dams: “El Cuchillo,” “Cerro Prieto,” and “La Boca” (Panel B in Figure 2) [10]. The remaining 40% is drawn from four key aquifers: “Área Metropolitana de Monterrey,” “Cañón del Huajuco,” “Campo Buenos Aires,” and “Campo Mina.” Water availability depends primarily on rainfall, so disruptions in the natural water cycle can seriously affect water supply for human domestic or hygiene needs [10].

The two joint climate stressors that led to the water crisis in 2022 were extreme drought and low precipitation. Since 2006, the state has faced increasing and severe droughts, reaching 96% of municipalities by April 2021 [11]. Precipitation levels in 2017 started to decrease below the historic mean (594 mm), attaining the lowest level in 2022 (~400 mm) [10]. Additionally, the four water reservoirs for domestic use in the MMA saw declining capacity in 2021, reaching historic lows between June and October 2022, particularly in “Cerro Prieto” reservoir, which dropped to 1% of its capacity [12]. Water infrastructure deficiencies aggravated the effects of climate stressors. On June 19, 2022, an aqueduct suffered a linear fissure [13]; its repair required closing the water pipeline, leaving 40% to 50% of the population without water supply for two days [13].

### Link between climate, water insecurity, and health

The climate stressors experienced by the MMA are observed globally. Between 2018 and 2022, several regions in the world broke historic records by enduring, on average, 47 days per person of health-threatening temperatures attributable to climate change [14]. The public health consequences of climate stressors are extensively documented. Increasing drought frequency has been linked to a 1.8% increase in food insecurity [14], and insect-borne diseases, like dengue, have nearly doubled each decade between 1990 and 2013 [15]. The fact that drought effects slowly accumulate over time makes it difficult to delineate their beginning and end [16], thus complicating [Bibr r16]the establishment of causal pathways between droughts and specific health outcomes [17]. Notwithstanding, a commonly identified pathway between extreme climate events and health outcomes is domestic water insecurity [18].

Household water insecurity is defined as the inability to reliably access and use water to meet basic domestic needs [19]. Water insecurity can be measured using the Water Insecurity Experience Scales (WISE) [20], which include questions about the frequency of problems related to water, such as experiences with water for consumption (e.g., drinking, cooking) and hygiene (e.g., handwashing), and consider psychological manifestations of water insecurity (e.g., worry, anger). In 2022, nationally representative data indicated that the prevalence of household water insecurity was 16% [21], but it was considerably higher in Nuevo Leon, at 43% [22].

Data on experiences with water insecurity using the WISE scales [18] have been used to estimate prevalence, monitor change, and reveal inequities [23]. Importantly, water insecurity has been linked to climate stressors; individuals from 29 countries who had experienced ≥1 month of floods or droughts were more likely to experience greater water insecurity [23]. Water insecurity has also shown direct and indirect connections with diverse health and economic outcomes, including physical and mental health, nutrition, well-being, and prosperity [18]. Water insecurity has consistently been associated with adverse health outcomes, including diarrhea [24], injury due to water collection [25], and gender-based interpersonal violence [26], and to sub-optimal nutrition [27], most notably food insecurity [28], but also lower dietary diversity [29] and infant and child feeding [30]. Moreover, household water insecurity has been linked to worse mental health [31], including higher stress [32], depression [33], and anxiety.

Household water insecurity is thus conceptualized as the mediator in the exposure pathway between climate events (i.e*.*, droughts and low precipitation) and nutrition, physical, and mental health outcomes. Both are moderated by the institutional and political context (i.e., the Nuevo Leon government and key stakeholders) and a social context characterized by a population with increased vulnerability (i.e., households in multidimensional poverty) [7]. This case study focuses on the state government’s response to mitigate the effects of a climate-related emergency on population well-being.

## Methods

The objective of the case study was to document post hoc lessons and challenges of the execution of the state’s emergency response to tackle household water insecurity. We relied on the EquIR [34] implementation science framework because it offers a conceptual lens for implementation of research in contexts characterized by poverty, institutional challenges, and limited capabilities.

We used three sources of information. The first was a review of academic and gray literature that broadly focused on water security in Mexico or Nuevo Leon during the period of the water crisis, namely between 2021 and 2024. We used the Web of Science to find academic literature using the terms “water insecurity” (and similar terms, like “water scarcity”), “Mexico,” and “Nuevo Leon.” For the gray literature, we used a similar strategy, but in Spanish, we searched in Google Scholar. The search was constrained to publications after the emergency, but we included references in selected publications when deemed relevant.

Second, we requested relevant documents describing the “Urban resilience in Nuevo Leon amid water scarcity” strategy from the Nuevo Leon Ministry of Equality and Social Inclusion (SII for its acronym in Spanish) and the state Office of Water and Sewage (SADM). We focused on actions by the SII because it was the agency responsible for implementing and coordinating with other actors the state government’s response to the water crisis. Third, we contrasted these documents with interviews with key informants who were directly involved in the implementation of the emergency response. These data sources helped identify the actions aimed at households and the facilitators and barriers to their implementation.

Identification of key informants occurred during a visit to Nuevo Leon in July 2023 to learn about experiences during the crisis with citizens, government officials, and members from private, academic, and social sectors [35]. The visit was an invitation from the SII to obtain technical assistance on using WISE scales to identify household water insecurity. The authors requested to carry out focus groups and in-person interviews with the mentioned stakeholders.

In 2024, twelve key informants were invited by email to participate in in-depth interviews: 10 out of 12 accepted. We did not receive a response from one private sector participant, and one staff member declined to participate, explaining that they did not consider they had the ability to respond. Interviewees were from the SII (*n* = 8), a high-level authority from SADM (*n* = 1), and the private sector (*n* = 1). Respondent roles were high-level decision-makers (*n* = 5) and staff members directly involved in the implementation of the adaptation strategies’ components (*n* = 5). The one-hour semi-structured interviews were carried out between July 5 and August 6, 2024, via Zoom, in Spanish, by the authors of the case study. All interviewees were informed of the study objectives, confidentiality measures were clarified, everyone granted oral consent to participate, and accepted the interview to be recorded. Interviews were transcribed with the Fireflies.ai software [36] and then de-identified for the analyses. The research project received an exempt status from the Universidad Iberoamericana’s Institutional Review Board (CEI-008-20160601).

We used a deductive approach to describe implementation outcomes from the EquIR, which are common categories in the implementation science literature [37, 38], used as guiding themes but operationalized to fit the characteristics of the government’s response during the crisis (Table 1). Thematic analyses followed a concept coding method [39]. De-identified interview transcripts were coded in a line-by-line approach by two researchers using a codebook which informed Table 1.

**Table 1:** Themes, definitions, operationalization of implementation outcomes, and excerpt inclusion criteria for each outcome.

IMPLEMENTATION OUTCOME/THEME	EQUIR DEFINITION	OPERATIONALIZATION	EXCERPT INCLUSION CRITERIA
Adoption	The intention or utilization of key elements for implementation	The initial decision and main mechanisms to execute the intervention	The excerpts referring to the intention, initial decision or action to try to employ a new intervention
Fidelity	Adherence of disadvantaged populations to the original intervention	How the intervention evolved and changed as challenges were encountered	Excerpts referring to the degree to which the intervention was implemented as it was designed in an original protocol, plan, or policy. In this emergency case, no such plans or protocols existed at the outset. This criterion captures the decision-making iterations that built the final response, highlighting the elements of improvisation and “learning as you go”
Cost	Financial and non-financial costs involved in the implementation of equity-focused elements	Elements of the intervention requiring goods, and human or financial resources	Excerpts referencing any goods involved in the delivery of the strategy; personnel, as well as mentions to the purchase or donation of goods and inputs, e.g. any mention of human and financial resources, number of goods such as water tanks, bottles, and liters
Acceptability	Satisfaction of stakeholders with the implementation	Reactions to the government strategy	Excerpts referencing the perception of stakeholders (e.g. consumers, providers, managers, policy-makers)
Coverage	Degree of reach, access, and coverage of the intervention among the disadvantaged population	Rationale to define the target population	Excerpts regarding the degree to which the population that was eligible to benefit from the strategy actually received it
Feasibility	Extent to which a program can be carried out in any setting, especially among disadvantaged populations	Contextual characteristics that resulted in variations in the implementation of the intervention	Excerpts mentioning the extent to which the strategy could be carried out in specific settings
Appropriateness	Relevance or perceived fit of the implementation in the disadvantaged population	Common barriers in the target population to using the intervention	Excerpts that mention the perceived fit or relevance of the intervention in a particular setting or for a particular target audience (e.g. provider or consumer)
Sustainability	Maintenance, continuation, or durability of the program through short, medium, and long-term strategies	Actions that originated during the emergency and remain in place	Excerpts referring to the extent to which elements of the intervention were maintained or institutionalized in a given setting

Discrepancies were handled first between the two coders; a third researcher gave his input to resolve the issues. All coders are authors of the case study. Analyses were conducted in Dedoose version 9.2.012, and a detailed account of selected quotes by informant role is available upon request.

## Findings

### Elements of a subnational emergency response to water insecurity

In Nuevo Leon, the severe water crisis led the subnational government to declare a state of emergency in February 2022 to access federal support and counteract the emergency. In March 2022, the state Office of Water and Sewage (SADM for its acronym in Spanish) alerted that some households were only receiving tap water two days a week or less. In response, the state government implemented emergency actions, including the “Urban resilience in Nuevo Leon amid water scarcity” [10].

**Figure 3:**
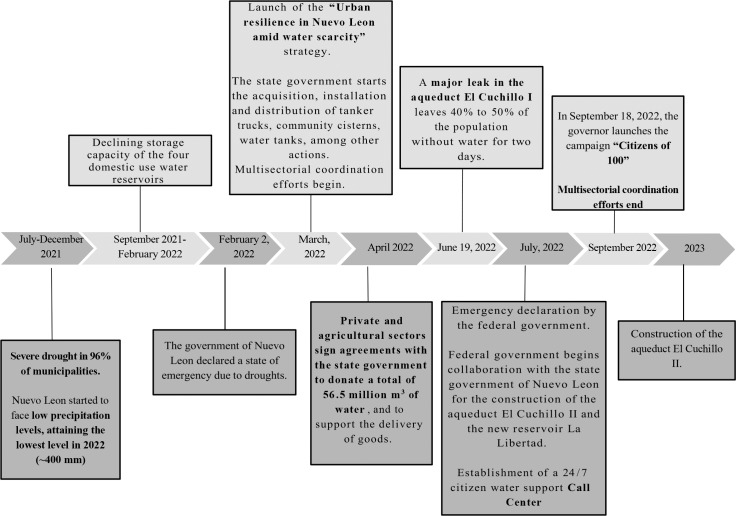
Timeline of the 2022 water crisis and ameliorative actions in the Monterrey Metropolitan Area (MMA).

The strategy mainly relied on the state’s disaster funds following the governor’s emergency declaration (see Figure 3) and, according to SADM, emergency actions were financed through a budget of 2,196 million MXP (approximately 115 million USD). Of this total, 58.4% came from SADM, 40.1% from other state government agencies, and 1.6% from the federal government. Most of this budget (45%) was allocated to the construction of deep wells, 19% to the acquisition of water trucks and community cisterns, 15% to pressure-regulating valves, 12% to the construction of shallow wells, 4% to the interconnection with some irrigation channels, 2% to dam pumping, and 2% to other actions [10].

Additional federal support to Nuevo Leon began in July 2022. The state and federal governments agreed to fund a new aqueduct and complete the construction of a reservoir to increase water availability [13, 40]. The federal government also deployed the Civil Relief and Aid Plan for Disasters (“Plan DN-III”), executed by the Ministry of Defense [3], to support the logistics of mitigation programs enacted by the state government.

The design and implementation of state programs directed to households were assigned to the Ministry of Equality and Social Inclusion (*SII* in Spanish), responsible for social policies to reduce poverty [41]. The SII coordinated multisectoral efforts between state agencies, with the federal government, and with other stakeholders (see Figure 3). The emergency response directed to the general population entailed intervention to reduce water insecurity in households or individuals; this intervention involved the SII handing out over 3,000 household water tanks (*tinacos*) with a capacity of 250 liters [42]; more than 500 community cisterns (*cisternas comunitarias*) of 5,000 and 10,000 liter capacity; delivered millions of liters of water with mobile tanker trucks (*pipas*); installed 400 “hydration points” to reduce the impact of heat waves [43]; distributed around 950,000 bottles of drinking water and close to 10,000 personal hygiene kits [42] (which included soap, menstrual hygiene supplies, and antibacterial gel); and the SII established a 24/7 citizen support call center, responsible for real-time monitoring of water distribution [43].

**Photo 1.**
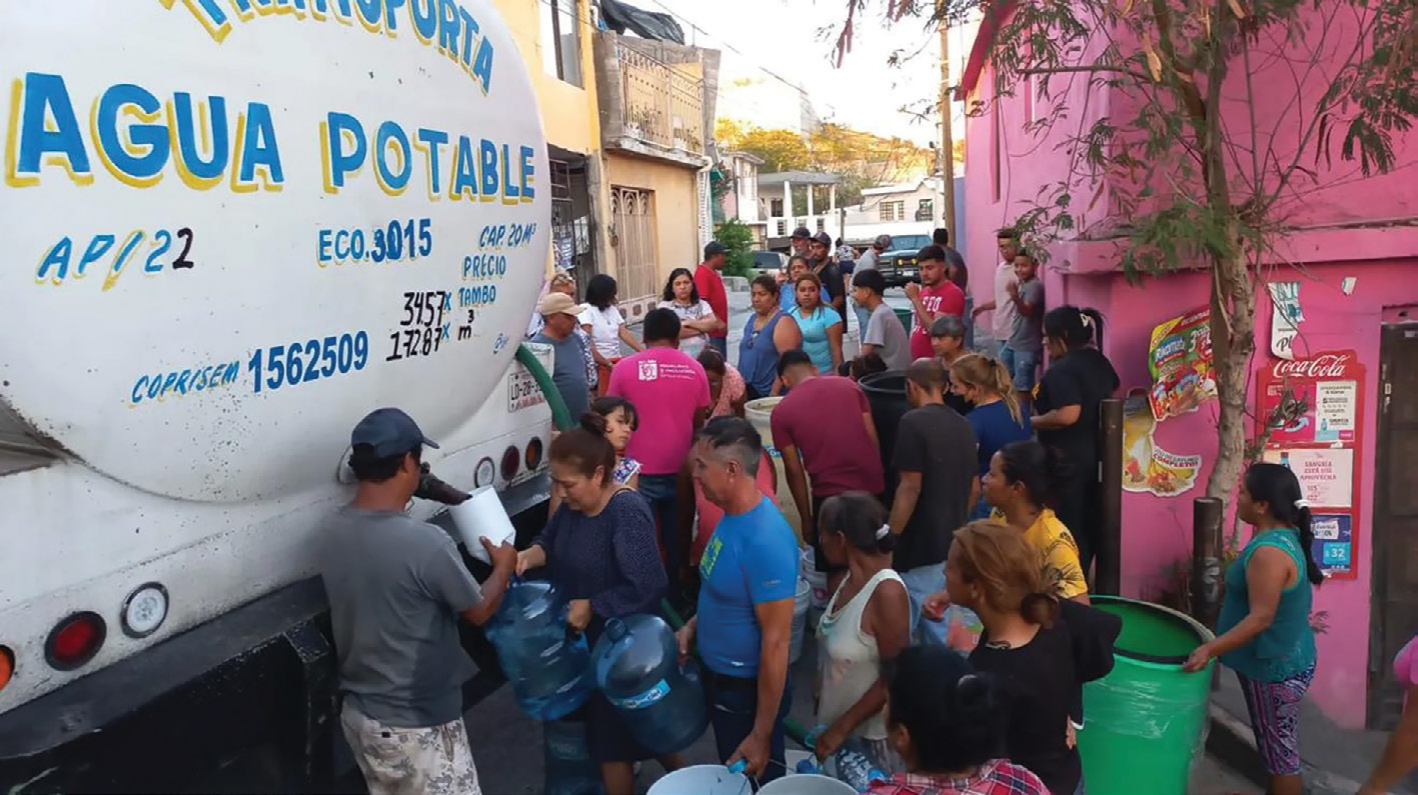
Families collecting water directly from a mobile tanker truck (*pipa*) in 2022. Credit: Daniel Rosales

The private and social sectors supported SII actions through outreach, donations, and the delivery of goods. Multiple private companies signed agreements with the subnational government to temporarily provide 21.8 m^3^ of water. The private sector complemented these actions by donating water bottles of multiple volumes (from 1.5 to 20 liters), electrolyte beverages, and personal hygiene kits (Photo 1). A beverage company produced more than 7 million cans of water, distributed by the SII, the Red Cross, and the Ministry of Defense. The industrial sector also committed to donate water trucks in rural areas for 36 months and farmers donated water for human consumption. Approximately, the industrial and agricultural sectors contributed 56.5 million m^3^ to supply water to the population [10]. Neighboring states sent mobile water tanks and drivers. According to SADM, collaboration among stakeholders benefited 1.8 million citizens of Nuevo Leon [10].

In addition, on September 18, 2022, the governor launched an awareness campaign, “Citizens of 100” *(Ciudadanos de 100*), to characterize how four-person households should use a maximum of 100 liters per day for daily activities [43, 44]. Historically, the average daily water consumption in Nuevo Leon ranged from 165 to 290 liters per person [10]. To meet the target of reducing usage to no more than 100 liters per person per day, the state government collaborated with the Ministries of Education, Culture, Citizen Participation, Tourism, Environment, the SII, Communications, and others to launch the “Water Culture Roundtable” (*Mesa de Cultura del Agua* in Spanish). This initiative brought together stakeholders to discuss strategies for reducing water use and preventing a future crisis [10].

### Implementation outcomes

#### Adoption

A key element in the adoption of the strategy was multi- and inter-sectoral cooperation. Logistics to implement the strategy began at the Social Cabinet, presided by the SII, oftentimes in the presence of the governor. The Social Cabinet is a pre-established high-level mechanism to coordinate inter-sectoral cooperation among state ministries. They created an emergency roundtable to coordinate the ground operation of the inter-sectoral response, which was led by SADM. Several actors became involved, such as personnel from the SII, the State’s Ministry of Education, the State Ministry of Health, and the governor’s office, among others. Between March and September 2022, these actors met daily at the roundtable to outline the specific elements of water delivery.

While SADM had the water availability monitoring system, they did not have direct contact with communities, and population needs exceeded their operative capacity during the shortage. This prompted SII’s role as a central agency in the deployment of the government’s response. Their mission as a government agency meant that they had data on the location of households in poverty, they coordinated community centers, and had a network of community members that served as liaisons (*enlaces ciudadanos*). Routes and target populations were defined at the roundtable and deliveries were carried out by SII personnel. Once the community cisterns were installed, liaisons would reach out to the call center and tanker trucks would be sent to fill them. The Nuevo Leon population played an important role in “communicating their needs” to better adapt the strategy.

Another example of cooperation was the use of private water well concessions. In Mexico, private companies are granted federal water concessions so they can extract water from wells for industrial purposes. During the state of emergency, private companies reached an agreement with the federal government in which certain industries ceded some of their rights to complement the water supply for human consumption. The partnership allowed the habilitation of additional wells to fill mobile water tanks, and private companies used their own water monitoring instruments to support the state government’s decision-making and policies.

#### Fidelity

There were no pre-existing plans that would constitute a response to a water shortage. The SII made key changes to the strategy as the crisis progressed. Initially, the strategy aimed to provide domestic water during the emergency. An important action was the deployment of tanker trucks (“*pipas*”), financed by public and private actors. People would approach the tanker truck with an array of containers, spanning from buckets, bottles, and a few devices with larger storage capacity; “people would come with their soda bottles and have them filled directly from the mobile water tanks” (see Photo 1). The disorder in water provision led to inefficiencies because water was lost during delivery.

**Photo 2.**
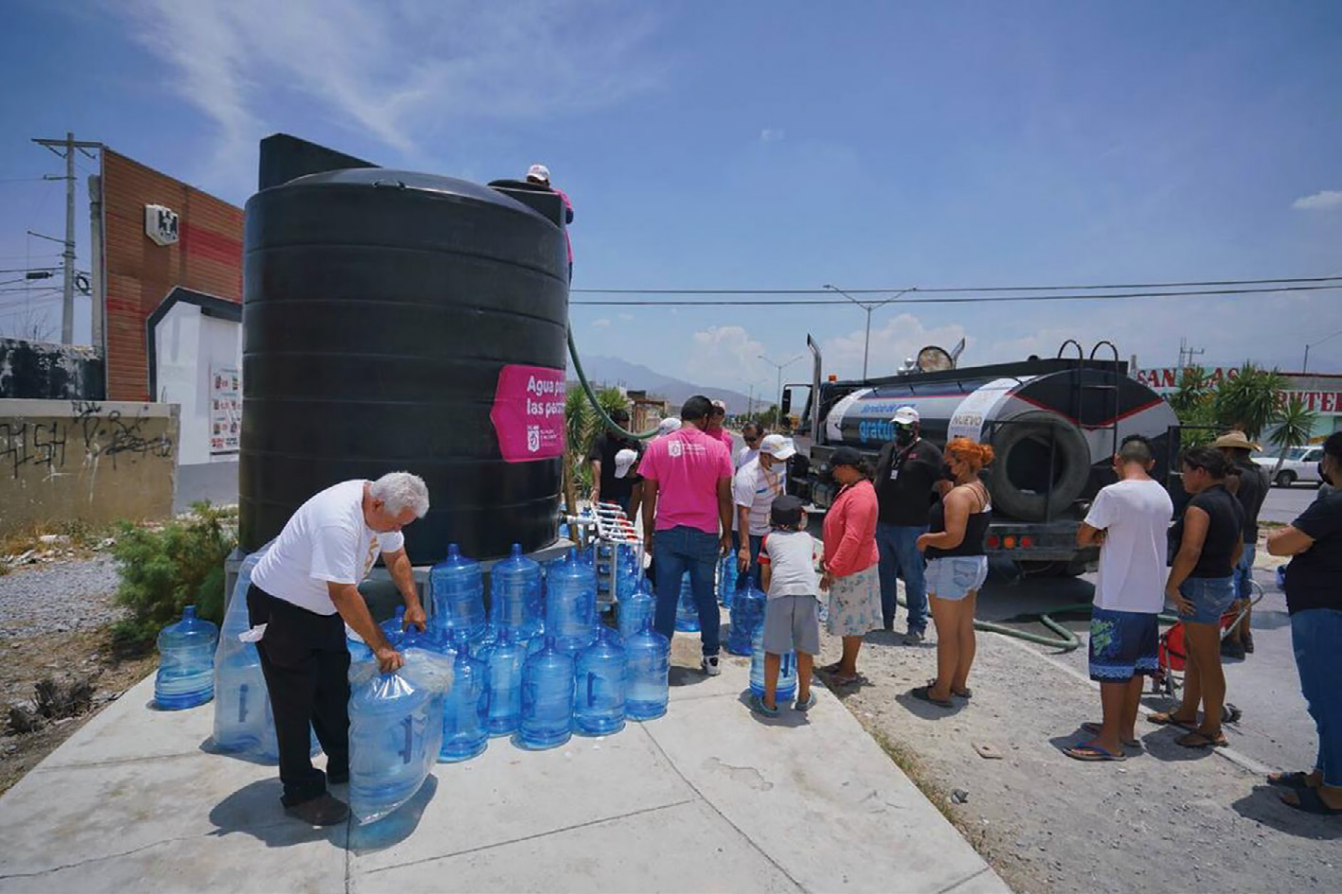
Water being collected from the community cistern after it has been filled with the mobile tanker truck. Credit: Daniel Rosales

Therefore, the SII decided to purchase and distribute household water tanks (*tinacos*) and install over 500 community cisterns (*cisternas comunitarias*) with a capacity of 5,000 and 10,000 liters in low-income neighborhoods. Water stopped being distributed directly from the “*pipa*” and it was used to fill community cisterns only (see Photo 2). Importantly, community leaders were appointed to coordinate water distribution, and then individuals would fill up their smaller household devices from the cistern. Moreover, the SII opened a 24/7 call center and *WhatsApp* line to receive citizen requests, address complaints, and monitor water allocation. Community liaisons would report directly to the call center if their water tank needed to be filled and SII would schedule a mobile water tank delivery. As the water shortage worsened, these actions were complemented by the distribution of donated water bottles, cans, and hygiene kits. Notably, informants did not mention a meaningful contribution from the organized civil society.

#### Cost

The specific cost analysis of the intervention was not done due to the extraordinary nature of its deployment. The emergency declaration allowed public purchases to be carried out without public bids, including geological studies and drilling for new wells. A process that usually takes a year occurred within just three months during this state of emergency. Dodging public bids also entails political risks because governments are more susceptible to being audited.

Respondents provided information on the sort of input involved in implementation. The SII had to purchase unanticipated equipment, such as water tanks and water bottles. Some of these items were donated by the private sector. A private company that yielded water rights estimated a donation of “3.7 million water liters of drinking water.” Mobile water tanks proved to be particularly cumbersome to hire, often coming from other states. Informants stressed that there were price fluctuations on water precisely because of its scarcity. This situation meant that the government had to buy large volumes of water, at higher prices, for a larger share of the population.

Another aspect that demanded higher costs was human resources because “additional personnel had to be hired at SII to meet the population’s demands.” A respondent who is involved in office work mentioned that they were responsible for a delivery route and that they would sometimes do fieldwork “from 7 AM to 9 PM, in the heat, and under harsh physical conditions.” These demands led to “teams frequently suffering from burnout.”

#### Acceptability

The intervention had to be implemented amid significant social discontent due to the lack of water for human consumption. Initially, the crisis triggered anxiety and anger among the population, “making it difficult to raise awareness and promote a sense of empathy between the communities and towards the personnel in charge”—who were also facing similar hardships at home.

Several respondents perceived that some communities were upset because they noticed that deliveries were prioritized: “The main challenge we faced was that we couldn’t tend to the entire population in one single day, it was gradual and often difficult to have certain communities to understand that.” Overall, respondents considered that the eligible population did end up benefiting from the strategy, and the challenge was figuring out *how* to meet the population’s needs in a timely manner.

The social discontent was most acute in the initial delivery of water tanks, when long waiting times, uncertainty in delivery schedules, and inefficient water containers angered the target population (see Photo 1). However, acceptability improved, and anger decreased once community tanks were in place, as they allowed people to organize themselves while guaranteeing a timely water supply (see Photo 2). 

#### Coverage

The dynamic process and constant expansion of targeting aid during the emergency precluded the SII from drafting a document with the exact coverage of their actions. SADM had a “general” distribution strategy, guided by water availability records and reports on the lack of water. In addition, it guaranteed that strategic facilities had sufficient water; for instance, they coordinated with the Ministry of Education the water supply at schools, and with the Ministry of Health to ensure hospitals, nursing homes, and other medical facilities met minimum operational water levels.

The SII followed a different approach by prioritizing the poorest communities. The SII already used the CHECS (*Cuestionario Homologado Estatal de Condciones Socioeconómicas* in Spanish) socioeconomic questionnaire as a targeting tool—used to estimate household poverty levels, conduct “territorial analyses,” and allocate social program benefits. The process was streamlined until the SII cross-referenced the CHECS poverty indicators with SADM community-level data to target water distribution actions.

While the SII was able to leverage its available tools to improve their crisis response, participants recognized that this crisis revealed additional needs, for which they had to expand their reach. One respondent explained they knew where informal settlements were located and how they usually struggled with regular water provision. However, this crisis also impacted social housing, where infrastructure was minimal and unfit to address these climate stressors.

#### Feasibility

Respondents mentioned several factors limiting the effectiveness of the response, especially among vulnerable populations. One of the main factors was the geographical characteristics, as the MMA is surrounded by high-altitude neighborhoods, oftentimes informal settlements. Reaching these areas to install the tanks “could take between 10 and 14 hours, from the SII to the community, depending on location and the level of difficulty to get there.” Once in those neighborhoods*,* it became difficult to settle the tanker trucks, particularly when they were filled with water, as they might weigh 10 tons, and the terrain made tank installation unfeasible.

Another compounding factor was population density. This meant that the demand for water varied across municipalities, and, consequently, the frequency with which a water tank needed to be sent to fill up varied as well: “in some communities, it was necessary to fill the community tank up to three times per day, while in others, we only needed to do it once a week.” Several respondents mentioned that a few mobile water tanks were intercepted en route. As a result, GPS was installed in the trucks and sometimes “tanker trucks and trucks with water bottles were escorted by the police in a huge operation”—as reported by a person involved in implementation. In some cases, the population would not allow empty trucks to leave the community until a replacement arrived.

#### Appropriateness

Respondents explained that the entire population of the MMA was affected, but some areas were worse off than others, particularly low-income neighborhoods that were at a further distance from the water reservoirs. As one informant reported: “Our efforts focused on the most vulnerable areas, which are the farthest from the water sources. Near the sources, they lacked water one day a week, maybe two. However, in the vulnerable areas, they could go a whole week without water*.*” The intervention included informal settlements, where houses were the least equipped and water service provision was precarious. Therefore, as an informant noticed, the emergency water provision was better than in their previous situation.

An important barrier was that houses did not have water storage infrastructure. Residents assumed that water would constantly flow in their faucets and there was no need to have storage containers. This limitation led the SII to distribute water tanks for houses and large buckets to transport water from the “pipas.” Moreover, respondents from the SII pointed out that their informal needs assessments with community liaisons showed that in some areas they had to provide wheeled carts, “*diablitos*,” to help citizens transport the water on steep streets. A concern emerged with water storage: mosquito-borne diseases, such as dengue. The SII collaborated with the Health Ministry to ensure the quality and innocuity of the water through chlorination in the community tanks and made sure they had lids. Communication campaigns reinforced the importance of keeping containers clean and closed.

#### Sustainability

Most of the actions of the intervention were rescinded once coordination efforts stopped and the emergency was subdued in September 2022 (see Figure 3). However, some community water tanks remained—those in informal settlements, approximately 20 out of the original 600 that were installed. The SII adopted its operation by providing mobile water tanks to keep filling them up. The inter-agency collaboration continued with the SADM because it proved to be a beneficial and useful complement: “They do not have the infrastructure to reach vulnerable communities, but the SII does.”

## Discussion

The government of Nuevo Leon, Mexico, dealt with the adverse consequences of a major water crisis in 2022 by ensuring household water security for the most vulnerable. They did this by securing water availability, increasing access for vulnerable populations, and by focusing on basic human consumption needs, such as drinking water. Notably, in a crisis with potential health and well-being consequences, the ministry that implemented the response was the SII, in charge of social policy, in a tightly coordinated real-time collaboration.

We were able to identify several facilitators for the implementation of the strategy. The emergency declaration was a policy instrument that helped raise awareness, provided additional funds, and removed bureaucratic barriers. The delimitation of a period of emergency energized stakeholders by increasing their willingness to collaborate. Coordination mechanisms such as the emergency roundtable proved essential for an integral response.

These mechanisms were sufficiently effective because these actors had already been working together, building trust. The Social Cabinet is an example of how previous inter-sectoral institutions were used and adapted for emergency response. Importantly, all goods and water provisions were free, both increasing water access to low-income households and assuaging price fluctuations due to scarcity. Likewise, the SII built a network of community liaisons who were a key link in the implementation process, and the regular interaction with their target population allowed them to report the community’s needs in real time. This operation was possible because the SII had a roster of social program beneficiaries, critical information on household poverty levels (CHECS), and its geographic location. Thus, the SII had the required social and data infrastructure and the flexibility to convert their regular activities for social program allocation into the needed activities to execute the government’s response to the crisis.

Several issues proved to be implementation challenges. The lack of preparedness was a cross-cutting issue. Interviewees believe the population lacked awareness of a potential water crisis, and most houses did not have cisterns or water tanks to store and transport water. However, the government had to quickly define a response, as there was no emergency plan for such a scenario. Initially, the response had to deal with widespread anger and anxiety due to the lack of water. Droughts were not an unpredictable phenomenon, and a stressed water infrastructure was prone to accidents, as the aqueduct’s fissure that resulted in a major leak at a critical moment (see Figure 3). Moreover, the immediate emergency response was unable to anticipate important barriers. Delivering water straight from the mobile water tanks was inefficient because precious water was lost in the process. The deployment of this action also angered the population because they were unable to plan and there was no established allocation mechanism. While the SII was able to correct these issues, it still faced additional hurdles, like the complicated terrain and increased water demands in high-density municipalities. While targeting had an equity rationale, it was not evident for the whole population. As one participant mentioned, “it is very difficult for the government to reach everyone…a government will never be enough for the whole population when it comes to meeting their needs or demands.” Notably, the private sector played an important role in the response, just as community leaders, but non-governmental social organizations were absent from their accounts, probably limiting the impact of the response.

Two unintended consequences stood out. First, the immediate water storage in households was not achieved in innocuous conditions. The reasons were an unhealthy reduction of water that may lead to disease and the increased risk of dengue in untapped water containers. The SII was able to coordinate with the Ministry of Health for water chlorination in the community tanks and to ensure that containers had lids. We could not find any epidemiological studies documenting the health consequences of these risks during the emergency; thus, we were unable to assess health impacts. The second unintended consequence was that the emergency response improved water service provision in informal settlements, mainly because it guaranteed an adjacent, reliable, and clean source of water. Only a few intervention actions remained after the emergency was under control. The SII was able to recognize this need in informal settlements and left the community water tanks that had been installed.

One key limitation of this study is the absence of beneficiary perspectives, which could offer relevant insights into health and hygiene risks during water shortages, such as climate-related diseases (e.g., dengue, diarrhea) and coping strategies (e.g*.*, the use of antibacterial gel or wet wipes). In informal settlements, such perceptions might have clarified whether the strategy effectively reduced perceived health risks, given pre-existing water conditions. In the specific context of an emergency response, we do not document sustainable actions such as those related to health system resilience [45, 46], which merit further research.

Relevant data helped target those most in need, especially low-income households without water—but other dimensions are still lacking, such as age, disability, and families with minors. The SII did not have an *ad hoc* early monitoring system, but after the crisis, they improved the CHECS by adding the WISE scales to their multidimensional poverty assessment. This will allow them to identify households that are already water-insecure when the next drought ensues. Notably, the crisis was framed as a water scarcity problem. Informants did not immediately link the climate stressors with health outcomes; rather, they focused on immediate basic needs such as water storage infrastructure. Efforts are needed to include program coverage and public health indicators in governments’ responses to climate change, which was not observed during the analyzed emergency response.

## Conclusions

Climate change will exacerbate water issues. Implementation Science can guide decision-makers, civil society, and researchers in presenting and prioritizing health adaptation and disaster risk management solutions, showing sustainability initiatives, and improving intervention strategies [47]. Allocating responsibilities for adaptation during climate-related emergencies can be challenging, as public health governance is often shared across levels and agencies, and reliable data may be unavailable. As this case study shows, state-level authorities are fundamental actors uniquely positioned to give an account of the implementation efforts, given their knowledge of their local population and their proximity to the impacts of the crisis [48].

The structured account of an intervention and its context, paired with the identification of barriers and facilitators, can contribute to underpinning the effective and sustainable implementation of public health interventions in real-world settings [49]. A description of the implementation of an intervention in Nuevo Leon helps identify blind spots that may improve with new iterations; for example, by adding preparedness components such as climate disaster reduction and prevention [50] because, as previously documented, drought mitigation efforts in Mexico have been focused on alleviation rather than prevention [51]. Lessons stemming from the case study can be useful to other cities facing similar threats [52] by showing ways to incorporate these strategies into their own social policies and avoid some of the known pitfalls. The case of Nuevo Leon points out how the combination of climatic stressors and infrastructure deficiencies, the population’s capacity to cope, and the government’s actions, all interact to shape the impacts of a crisis. Successful mitigation strategies may stem from strengthening inter-sectoral collaboration and improved monitoring systems for decision-making. The Nuevo Leon case study will surely nurture the scientific landscape with accounts uncovering the links between water insecurity and health.
